# Supplementary material for: A high-resolution mRNA expression time course of embryonic development in zebrafish
Source: eLife. 2017 Nov 16;6:e30860. doi: 10.7554/eLife.30860 (PMC5690287; doi:10.7554/eLife.30860)
Supplement: Supplementary file 6. [file elife-30860-supp6.zip › biolayout-clusters-files/Cluster239-genes.html]

Cluster239


# Cluster239: Genes

| Ensembl ID | Gene Name | Chr | Start | End | Biotype |
| --- | --- | --- | --- | --- | --- |
| ENSDARG00000087887 | PHF21B | 4 | 73251704 | 73485393 | protein\_coding |
| ENSDARG00000058259 | celsr1b | 25 | 19655984 | 19767158 | protein\_coding |
| ENSDARG00000070337 | hoxc1a | 23 | 36079301 | 36092185 | protein\_coding |
| ENSDARG00000089435 | macf1b | 16 | 5680118 | 5683138 | protein\_coding |
| ENSDARG00000052094 | notch1b | 5 | 64998482 | 65103741 | protein\_coding |
| ENSDARG00000100829 | pcdh1g32 | 10 | 21850116 | 21862676 | protein\_coding |
